# Supplementary material for: Single molecule compression reveals intra-protein forces drive cytotoxin pore formation
Source: eLife. 2015 Dec 10;4:e08421. doi: 10.7554/eLife.08421 (PMC4714976; doi:10.7554/eLife.08421)
Supplement: Supplementary file 1. — The proteins were aligned using BLAST. Shown in parentheses are the residue numbers for each protein. DOI: http://dx.doi.org/10.7554/eLife.08421.016 [file elife-08421-supp1.docx]

| Perfringolysin O | Phe75 | Tyr320 | Tyr415 |
| --- | --- | --- | --- |
| Phaericolysin | Phe (81) | Tyr (397) | Tyr (423) |
| Anthrolysin O | Phe (88) | Tyr (402) | Phe (428) |
| Cereolysin O | Phe (88) | Tyr (402) | Phe (428) |
| Thuringiensilysin O | Phe (88) | Tyr (402) | Phe (428) |
| Weihenstephanensilysin | Phe (88) | Tyr (402) | Phe (428) |
| Alveolysin | Phe (77) | Tyr (391) | Tyr (417) |
| Brevilysin | Phe (85) | Tyr (400) | Tyr (426) |
| Butyriculysin | Phe (89) | Tyr (401) | Tyr (427) |
| Tetanolysin O | Phe (101) | Tyr (415) | Tyr (441) |
| Botulinolysin B | Tyr (176) | Tyr (490) | Tyr (516) |
| Botulinolysin E3 | Tyr (176) | Tyr (490) | Tyr (516) |
| Botulinolysin C | Tyr (93) | Tyr (407) | Tyr (433) |
| Novyilysin | Tyr (89) | Tyr (403) | Tyr (429) |
| Streptolysin O | Phe (146) | Tyr (460) | Tyr (486) |
| Streptolysin O c | Phe (149) | Tyr (463) | Tyr (489) |
| Streptolysin O e | Phe (146) | Tyr (460) | Tyr (486) |
| Lectinolysin | Phe (240) | Tyr (552) | Tyr (578) |
| Pneumolysin | Phe (43) | Tyr (358) | Tyr (384) |
| Mitilysin | Phe (43) | Tyr (358) | Tyr (384) |
| Pseudopneumolysin | Phe (43) | Tyr (358) | Tyr (384) |
| Suilysin | Phe (70) | His (386) | Tyr (412) |
| Intermedilysin | Tyr (101) | Tyr (416) | His (442) |
| Ivanolysin | Tyr (97) | Tyr (413) | Tyr (439) |
| Seeligeriolysin O | Tyr (99) | Tyr (415) | Tyr (441) |
| Listeriolysin O | Tyr (98) | Tyr (414) | Tyr (440) |
| Vaginolysin | Phe (88) | Tyr (403) | Tyr (427) |
| Pyolysin | Tyr (103) | Tyr (422) | Tyr (448) |
